# Coding Variation and Adherence to Methodological Standards in Cardiac Research Using the National Inpatient Sample

**DOI:** 10.3389/fcvm.2021.713695

**Published:** 2021-11-02

**Authors:** John W. Ostrominski, Javier Amione-Guerra, Brian Hernandez, Joel E. Michalek, Anand Prasad

**Affiliations:** ^1^Department of Medicine, Division of Cardiology, UT Health San Antonio, San Antonio, TX, United States; ^2^Department of Population Health Sciences, UT Health San Antonio, San Antonio, TX, United States

**Keywords:** administrative datasets, HCUP, national inpatient sample (NIS), ICD-9-CM (international classification of diseases. 9th revision. clinical modification), acute myocardial infarction

## Abstract

**Background:** Code selection is crucial to the accuracy and reproducibility of studies using administrative data, however a comprehensive assessment of coding trends for major cardiac diagnoses and procedures is lacking. We aimed to evaluate trends in administrative code utilization for major cardiac diagnoses and procedures, and adherence to required methodological practices in cardiac research using the National Inpatient Sample (NIS).

**Methods:** In this observational study of 445 articles, ICD-9-CM codes corresponding to acute myocardial infarction (AMI), heart failure, atrial fibrillation, percutaneous coronary intervention, and coronary artery bypass grafting were collected and analyzed. The NIS was used to compare the number of hospitalizations between the most frequently encountered AMI case definitions. Key elements were abstracted from each article to evaluate adherence to required methodological practices.

**Results:** Variation in code utilization was observed for each diagnosis and procedure assessed, and the number of unique case definitions published per year increased throughout the study period (*P* < 0.001), driven largely by the significant increase in articles per year (*P* < 0.001). Off-target codes were observed in 39 (8.8%) studies. Upon reintroduction into the NIS for 2008–2012, the most commonly encountered case definitions for AMI were found to yield significantly different estimates of AMI hospitalizations and hospitalization trends over time. Three hundred and ninety-nine articles (84%) did not adhere to one or more required research practices. Overall adherence was superior for publications in higher-impact journals (*P* = 0.002).

**Conclusions:** Substantial variation in code selection exists for major cardiac diagnoses and procedures, and non-adherence to methodological standards is widespread. These data have important implications for the accuracy and generalizability of analyses using the NIS.

## Introduction

Administrative datasets constitute an important source of information for “big data” applications in cardiovascular medicine (1), however their analytic value is predicated upon the use of research methods that are accurate and reproducible (2). As highlighted by recent national-level performance measure and quality improvement guidelines, selection of appropriate administrative codes is imperative for the identification of target populations for disease or procedure-specific analyses (3–5). Divergences in code selection have the potential to confound epidemiologic estimates, outcome measures, and cost/value determinations.

The National Inpatient Sample (NIS), sponsored by the Agency for Healthcare Research and Quality (AHRQ) as part of the Healthcare Utilization Project (HCUP), is the largest all-payer administrative database and includes data from a 20% stratified sample of hospitalizations in participating states (*N* = 47), representing more than 97% of the United States population (6). Owing to the large sample size, coupled with its ease and low cost of access, NIS utilization has outpaced that of other administrative databases (2). However, concerns remain regarding the ability of researchers to manage its specific methodological challenges (2, 7, 8). It was recently shown that non-adherence to methodological practices required by the AHRQ was prevalent in a representative sample of studies employing the NIS, and that such deviations have the potential to impact study conclusions (9).

Despite the importance of code selection in research efforts using the NIS, a contemporary analysis of coding trends for major cardiac diagnoses and procedures is lacking. The purpose of the present study was to evaluate the consistency of International Classification of Diseases, Ninth Revision—Clinical Modification (ICD-9-CM) code reporting for several major cardiac diagnoses and procedures, as well as to analyze adherence to required methodological standards in cardiac research using the NIS.

## Methods

### Literature Search and Study Selection

We queried PubMed, CINAHL, Scopus, Medline, and the Healthcare Cost and Utilization Project (HCUP) Publication Search for all publications using the NIS and principally focused on any element of cardiac disease. The analysis period allowed only for the collection of ICD-9-CM codes. The following electronic search strategy was used, from inception to March 1st, 2018: ((“National Inpatient Sample” [all fields] OR “Nationwide Inpatient Sample” [all fields]) AND (“heart” [title/abstract] OR “cardiac” [title/abstract] OR “coronary” [title/abstract] OR “bypass graft” [title/abstract] OR “infarction” [title/abstract] OR “valve” [title/abstract] OR “stent” [title/abstract] OR “percutaneous coronary intervention” [title/abstract] OR “arrhythmia” [title/abstract] OR “fibrillation” [title/abstract] OR “flutter” [title/abstract] OR “atrial” [title/abstract] OR “ventricular” [title/abstract] OR “endocardium” [title/abstract] OR “myocardium” [title/abstract] OR “epicardium” [title/abstract])). Articles examining extra-cardiac structures or non-cardiac procedures were excluded from the study. Individual search terms were adapted for the syntax of each database, and no language restrictions were applied.

One researcher (J.W.O.) evaluated article eligibility based on the titles and abstracts of all studies identified in the electronic search. References of each full-text study included in the qualitative analysis were independently reviewed by two researchers (J.W.O. and J.A.G.) for additional relevant articles. Discrepancies were resolved by consensus among the study team.

### Data Extraction

Data were abstracted from each of the included articles *via* distribution of standardized data collection forms to each investigator, outlining eligibility criteria, pre-specified major cardiac diagnoses and procedures, analyses, and other relevant study details. Journal Citation Reports (10) (JCR) was used to determine journal impact factor. Journals not listed within JCR were assigned an impact factor of zero.

### Code Collection and Analysis of Coding Trends

During full-text article review, two independent investigators (J.W.O. and J.A.G) recorded ICD-9-CM codes if reported for each of the pre-specified cardiac diagnoses or procedures: acute myocardial infarction (AMI), heart failure (HF), atrial fibrillation (AF), percutaneous coronary intervention (PCI), and coronary artery bypass grafting (CABG). If specified by authors, codes corresponding to ST-elevation myocardial infarction (STEMI), non-ST elevation myocardial infarction (NSTEMI), or unstable angina (UA) were also recorded. Codes were compiled and reviewed for accuracy with the assistance of a professional medical coder. To diminish the impact of variation in study question as a source of coding variability, codes were excluded from the study if authors either (1) reported specifiers that would engender the use of particular code sets, such as “multivessel PCI” or (2) explicitly excluded specific diagnosis or procedure codes. Frequencies of individual codes and groups of codes (case definitions) used to define diagnoses and procedures were computed. To enable quantitation and analysis of individual code trends, codes summarized as a range, with a “.x” or “.xx” (such as 428.x or 428.xx), or as Clinical Classifications Software (CCS) codes (11), were separated into their constituent codes. Where applicable, extraneous codes not found in the CMS database (12) (e.g., 428.5, 428.6, 428.7, and 428.8) were removed.

### Assessment and Definition of Off-Target Coding

Codes cited for each of the prespecified diagnoses and procedures also underwent further adjudication to ensure concordance between the reported diagnosis or procedure of interest, and the codes used. This was performed by comparing the codes extracted from studies with those in the CMS database (12). Coding was labeled “off-target” when reported ICD-9-CM codes were discordant with their intended diagnosis or procedure (e.g., inclusion of codes for unstable angina when the intended diagnosis was AMI).

### Analysis of AMI Discharge Counts Using Case Definitions Extracted From the Literature

To determine the impact of the observed coding variation upon hospitalizations, we selected AMI as a representative diagnosis and employed the NIS to compute the number of hospitalizations per year for the most frequently encountered case definitions for AMI, using the 2008–2012 data years. Specifically, we used (1) [4100, 4101, 4102, 4103, 4104, 4105, 4106, 4107, 4108, 4109], (2) [41000, 41001, 41002, 41010, 41011, 41012, 41020, 41021, 41022, 41030, 41031, 41032, 41040, 41041, 41042, 41050, 41051, 41052, 41060, 41061, 41062, 41070, 41071, 41072, 41080, 41081, 41082, 41090, 41091, 41092], (3) [4100, 4101, 4102, 4103, 4104, 4105, 4106, 4107, 4108, 4109, 41000, 41001, 41002, 41010, 41011, 41012, 41020, 41021, 41022, 41030, 41031, 41032, 41040, 41041, 41042, 41050, 41051, 41052, 41060, 41061, 41062, 41070, 41071, 41072, 41080, 41081, 41082, 41090, 41091, 41092], (4) [410], (5) [41000, 41001, 41010, 41011, 41020, 41021, 41030, 41031, 41040, 41041, 41050, 41051, 41060, 41061, 41070, 41071, 41080, 41081, 41090, 41091], (6) [41011, 41021, 41031, 41041, 41051, 41061, 41071, 41081, 41091], (7) [410, 411], and (8) [4100, 4101, 4102, 4103, 4104, 4105, 4106, 4108, 4109]. In the first model, the number of hospitalizations generated by the most common case definitions used to establish AMI as a primary diagnosis (#1–6)—the diagnosis chiefly responsible for the hospital admission—were compared. In the second model, the number of hospitalizations generated by common case definitions used to establish AMI as a secondary diagnosis (#1, 7, and 8)—diagnoses that are either comorbid at the time of admission or develop during the admission—were compared. In each model, hospitalizations with age < 18 were excluded. All statistical analyses for the data simulations were performed using the trend weights provided by the AHRQ, accounting for the revision in NIS data structure in 2011–2012 (13). A logistic linear model using SAS PROC GENMOD was constructed to account for hospital clustering. The relative risk (RR) and 95% confidence interval (CI) for the RR is reported. The significance of temporal trends was assessed with the slope (β) and its standard error (SE) in a log linear model.

### Evaluation of Adherence to AHRQ Methodological Standards

To assess whether the study population of articles adhered to required research practices, a protocol was adapted from previously reported methods (2, 9, 14). Briefly, articles were systematically evaluated for adherence to seven major methodological practices required by the AHRQ in research using the NIS: (1) identification of observations as inpatient events rather than individual patients (15), (2) avoidance of analyses that inappropriately estimate state-specific statistics (16), (3) avoidance of hospital volume analyses after 2011 (13), (4) avoidance of analyses that inappropriately estimate statistics for individual physicians (17), (5) avoidance of secondary diagnosis codes that fail to distinguish between complications and comorbidities (non-specific codes) (18), (6) utilization of statistical methods, including hierarchical or mixed-effects models, to account for the complex survey structure of the NIS (19, 20), and (7) adjustment for major transition periods in NIS sampling technique for analyses concerning temporal trends (13, 21). Two investigators (J.W.O. and J.A.G.) independently reviewed the studies and tabulated adherence to each of the aforementioned research practices. Discrepancies were resolved through mutual agreement.

### Statistical Methods

In the literature analysis, continuous variables were summarized as median (range), and categorical variables expressed as frequencies paired with percentages. The χ^2^ and Fisher Exact test with Bonferroni correction were used for comparison of differences in categorical outcomes. To evaluate trends of case definition publication over time, case definitions and articles were tabulated by year (1998–2017) and cardiac diagnosis/procedure (AF, AMI, CABG, HF, PCI). The significance of variation in case definitions with year and diagnosis/procedure was assessed with a Generalized Estimating Equations (GEE) Poisson model with a log link of cases in terms of year, diagnosis/procedure, and the year by diagnosis/procedure interaction with PCI as the referent type. Variation in articles was assessed similarly. A combined analysis of cases in terms of articles, year, and diagnosis/procedure was also carried out with articles entered as a numerical covariate and including all pairwise interactions. All analyses were performed using SAS 9.4 (SAS Institute), 2-sided statistical tests, and a significance threshold of *a* = 0.05. This use of NIS data for this study was exempted from further consideration by the UT Health San Antonio's institutional review board as the data are de-identified.

## Results

**Figure 1:**
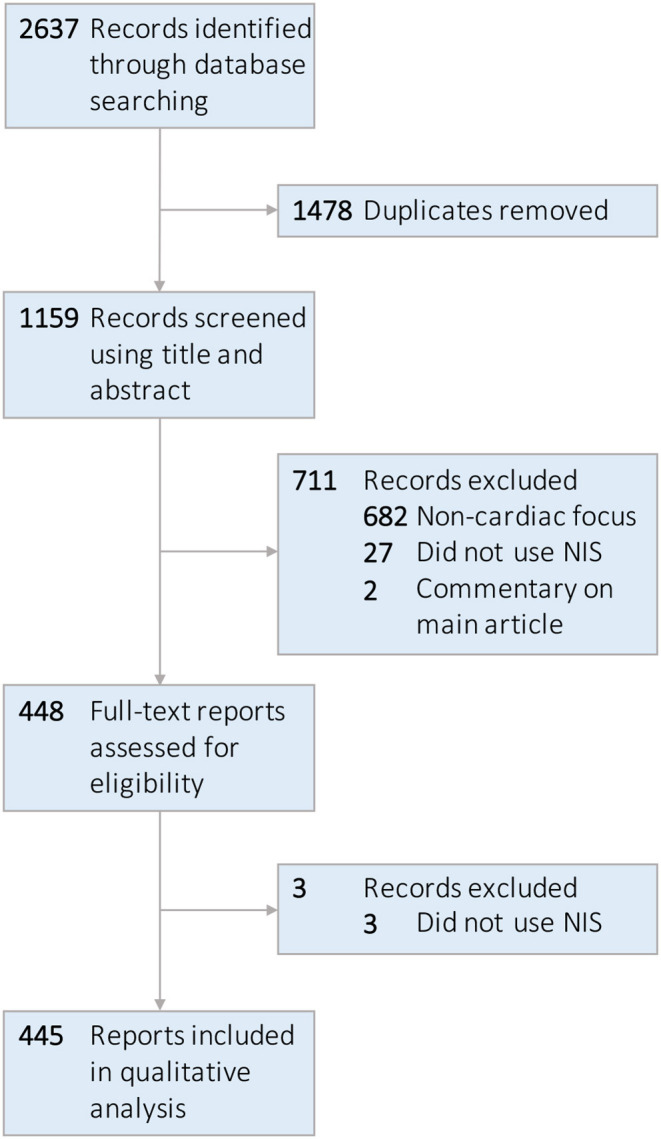
Flow diagram of study selection process.

The electronic literature search retrieved 2,637 articles. Following removal of 1,478 duplicates, one reviewer (J.W.O.) screened 1,159 unique studies for eligibility *via* assessment of title and abstract. 448 full-text articles underwent review, after which an additional 3 were excluded due to lack of utilization of the NIS, yielding 445 studies eligible for inclusion in the observational study. No additional articles were uncovered after reference review. A graphical representation of the study selection procedure is presented in Figure 1.

### Selected Descriptive Characteristics of Included Studies

**Figure 2:**
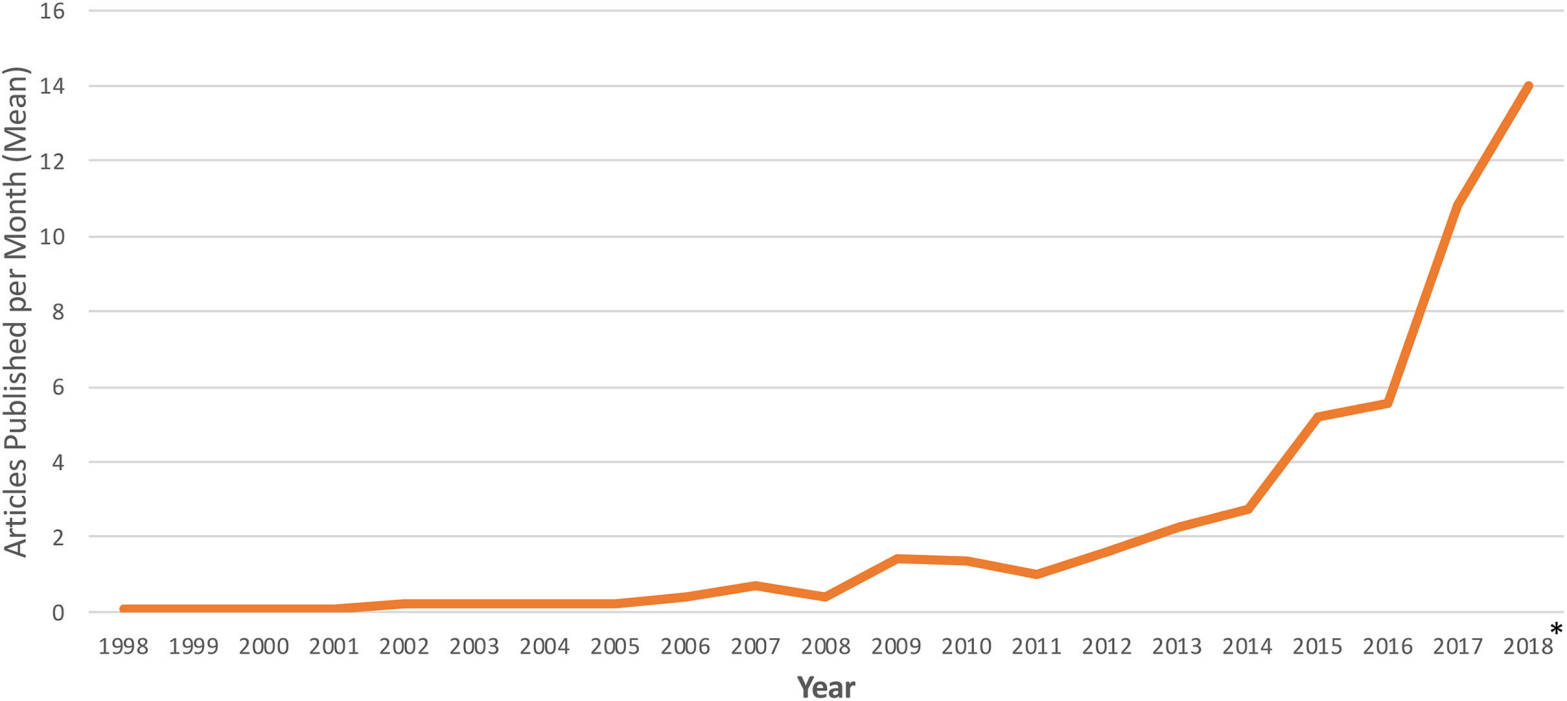
Temporal trends in publication of cardiac research using the national inpatient sample. Frequency of NIS utilization is reported as number of articles published per month. *Represents average number of articles per month for January 1st, 2018 through March 1st, 2018.

Studies meeting inclusion criteria spanned from 1998 to 2018 and featured a median interval of 8 data years (range 1–18). NIS utilization in cardiac research has increased dramatically, with 175-fold more studies published per month in 2018 compared to 1998 (Figure 2). 2008 was the most frequently encountered data year in the study cohort and included in 269 (60.5%) articles. The articles included in the study were published in journals with a median impact factor of 3,400 (range 0–44.405), and 24 (5.4%) were published in journals with an impact factor greater than or equal to 10.

### Coding Trends for Major Cardiac Diagnoses and Procedures

Of the 445 articles included in the study, 435 (97.8%) reported specific ICD-9-CM diagnosis or procedure codes. 123 (27.6%) studies reported codes for AMI, 158 (35.5%) reported codes for HF, 63 (14.2%) reported codes for AF, 94 (21.1%) reported codes for PCI, and 134 (30.1%) reported codes for CABG. With respect to acute coronary syndrome subtypes, 43 (9.7%), 26 (5.8%), and 10 (2.2%) studies specifically reported codes for STEMI, NSTEMI, and UA, respectively.

In the study population of articles, substantial variation in ICD-9-CM code selection was observed for each of the pre-specified diagnoses and procedures. These findings are summarized in Table 1. Across the study cohort of articles, 21 unique case definitions were observed for AMI, and similar trends were observed for HF (37 case definitions), PCI (37 case definitions), and CABG (23 case definitions). Despite the existence of only one ICD-9-CM code for AF (12), 5 different case definitions were observed during literature review. Among articles published in journals with an impact factor of 10 or greater, variation in code selection was also observed for AMI, HF, PCI, and CABG.

**Table 1:** Unique ICD-9-CM codes and case definitions for major cardiac diagnoses and procedures in cardiac research using the national inpatient sample.

**Cardiac diagnosis or procedure**	**Articles reporting codes (*n* = 445) No. (%)**	**Unique individual codes No**.	**Codes per case definition median (Range)**	**Unique case definitions: impact factor < 10 No.**	**Unique case definitions: impact factor ≥ 10 No.**
Acute myocardial infarction	123 (27.6)	49	10.0 (1–40)	21	5
ST-segment elevation myocardial infarction	43 (9.7)	41	9.0 (1–39)	17	-
Non-ST-segment elevation myocardial infarction	26 (5.8)	9	2.5 (1–6)	10	-
Unstable angina	10 (2.2)	5	1.5 (1–4)	5	-
Heart failure	158 (35.5)	60	10.5 (1–29)	37	7
Atrial fibrillation	63 (14.2)	3	1.0 (1–3)	5	1
Percutaneous coronary intervention	94 (21.1)	21	5.0 (1–12)	37	6
Coronary artery bypass grafting	134 (30.1)	23	9.0 (1–21)	23	5

**Figure 3:**
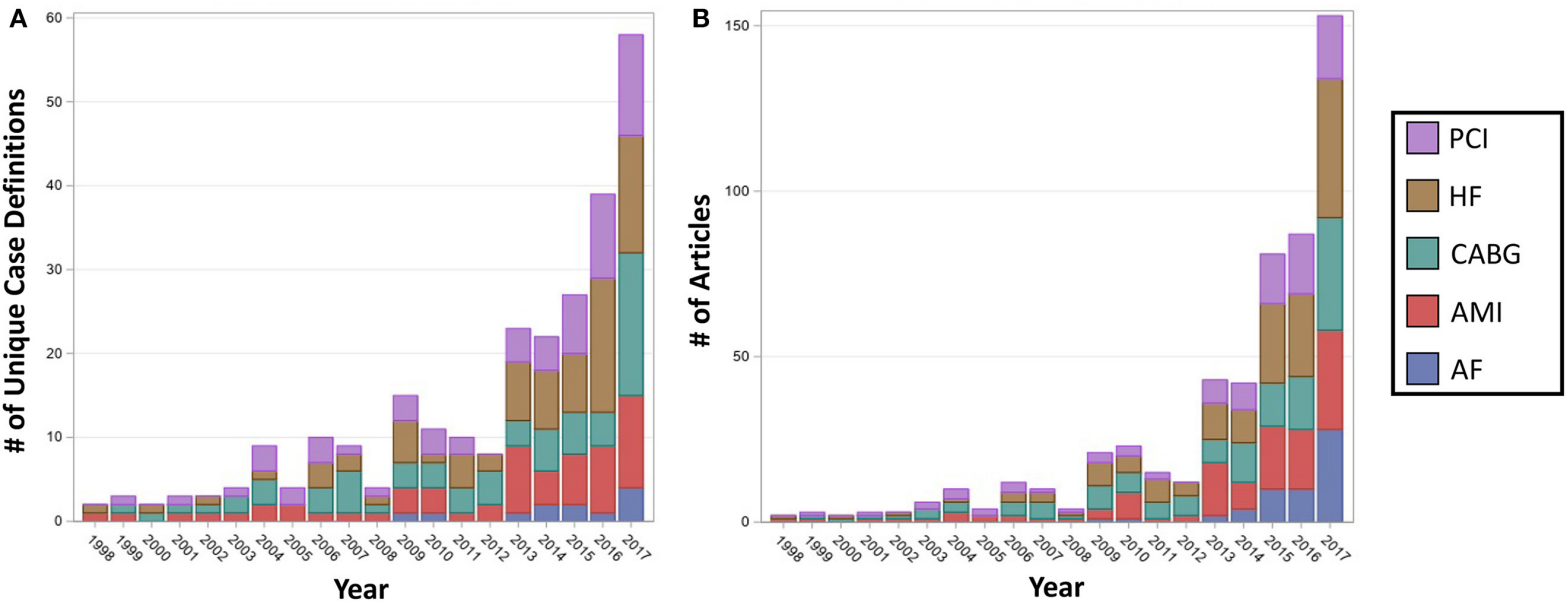
Number of unique case definitions and articles published per year in cardiac literature using the national inpatient sample, by cardiac diagnosis or procedure. The number of unique case definitions **(A)** and articles **(B)** were found to be significantly increasing with year for each of the cardiac diagnoses and procedures (*P* < 0.001). Overall, the number of unique case definitions per year was found to increase with the number of articles published per year. After adjustment for the number of articles per year, the number of unique case definitions per year was found to decrease with time (*P* < 0.001). AF, Atrial Fibrillation; AMI, Acute Myocardial Infarction; CABG, Coronary Artery Bypass Grafting; HF, Heart Failure; PCI, Percutaneous Coronary Intervention.

In an analysis of temporal trends, the number of unique case definitions reported per year was found to be increasing throughout the study period for all cardiac diagnoses and procedures assessed (*P* < 0.001) (Figure 3A). There was no significant association between the number of unique case definitions per year and cardiac diagnosis/procedure over time (median number of case definitions per year, AF 0, AMI 2, CABG 3, HF 2, PCI 2; *P* = 0.11). The number of articles published per year also substantially increased (*P* < 0.001) (Figure 3B) and varied significantly by cardiac diagnosis/procedure (median number of articles per year, AF 0, AMI 2, CABG 5, HF 3, PCI 2; *P* < 0.001), likely due to the increased prevalence of CABG and HF articles in recent years. Overall, the number of unique case definitions per year was found to increase with the number of articles published per year. After adjustment for the number of articles per year, the number of unique case definitions per year was found to decrease with time (*P* < 0.001).

### Off-Target Coding

Upon adjudication of the constituent codes for each case definition, off-target codes were detected for each of the cardiovascular diagnoses and procedures assessed. In total, 39 (8.8%) articles, comprising 6.1% of all case definitions (*n* = 651) examined, included one or more off-target codes. These data are summarized in Table 2. The prevalence of off-target coding was higher for diagnoses vs. procedures (8.3 vs. 1.8%, *P* = 0.001) across the study period. Off-target coding for each of the prespecified diagnoses or procedures was not observed in articles published in journals with an impact factor of 10 or greater.

**Table 2:** Prevalence of off-target ICD-9-CM codes for major cardiac diagnoses and procedures in cardiac research using the national inpatient sample.

**Cardiac diagnosis or procedure**	**Unique individual codes Total No**.	**Off-target codes No. (% of total codes)**	**Articles citing off-target codes No. (% of articles)**
Acute myocardial infarction	49	8 (16.3)	8 (6.5)
ST-segment elevation myocardial infarction	41	4 (9.8)	5 (11.6)
Non-ST-segment elevation myocardial infarction	9	5 (55.6)	6 (23.1)
Unstable angina	5	2 (40.0)	3 (30.0)
Heart failure	60	28 (46.7)	7 (4.4)
Atrial fibrillation	3	2 (66.7)	6 (9.5)
Percutaneous coronary intervention	21	1 (4.8)	2 (2.1)
Coronary artery bypass grafting	23	3 (8.7)	2 (1.5)

*Prevalence of off-target coding in the study cohort is displayed as (1) off-target codes as a percentage of all unique codes encountered for each diagnosis or procedure (column 3), and (2) number of articles citing off-target codes as a percentage of all articles citing codes for that diagnosis or procedure. All articles citing off-target codes were published in journals with impact factor < 10*.

Across the population of articles reporting codes for AMI, 8 (6.5%) studies included one or more off-target codes in their case definitions. Specifically, we observed the inclusion of codes for angina, as well as those for acute or subacute coronary syndromes without myocardial infarction. Codes referring to subendocardial infarction/NSTEMI were also present in 5 (11.6%) of all studies reporting codes for STEMI, whereas derivatives of the parent code “4109,” referring to STEMI per an ICD-9-CM revision in 2005 (22), was present in 6 (23.1%) NSTEMI articles. Importantly, each of the studies employing this code were published after 2005. Similar trends were observed for UA, wherein 3 (30%) of articles included at least one off-target code.

Off-target codes were reported by 7 (4.4%) articles reporting codes for HF, comprising 51.7% of the 60 unique ICD-9-CM codes observed. These referred to various forms of cardiomyopathy, dyspnea, acute pulmonary edema, acute coronary syndrome, or even viral pneumonia (“480xx”). Additionally, we also observed “39881,” which was not present in any iteration of the CMS ICD-9-CM database (12). Finally, off-target codes were observed in 6 (9.5%) articles reporting codes for AF, representing atrial flutter (6.3%) or the parent code for atrial fibrillation and flutter (4.8%). With respect to procedural codes, 2 (2.1%) articles cited “366” in their PCI case definitions, however this was also not observed in the CMS database of procedure codes. Lastly, off-target codes were observed in 2 (1.5%) articles citing CABG codes, referring to extracorporeal circulation, systemic hypothermia, or PCI.

Coding schema developed by the HCUP for disease- or procedure-based analyses (11) were poorly represented, consisting of 12.8% of all eligible case definitions (AMI, HF, PCI, or CABG; *n* = 509). Case definitions corresponding to established comorbidity measures (23–25) were more frequently encountered, comprising 27.6% (34/123) of AMI and 51.9% (82/158) of HF case definitions. Finally, case definitions concordant with extant quality improvement guideline recommendations (3–5, 26–28) were infrequently encountered for both AMI and HF, reported in only 8.9 and 1.3% of articles, respectively. No difference was observed between high- vs. low-impact publications (*P* = 0.750) in reporting of case definitions presented in quality improvement guidelines.

### Comparison of AMI Discharge Counts by Unique Case Definition in the NIS

**Figure 4:**
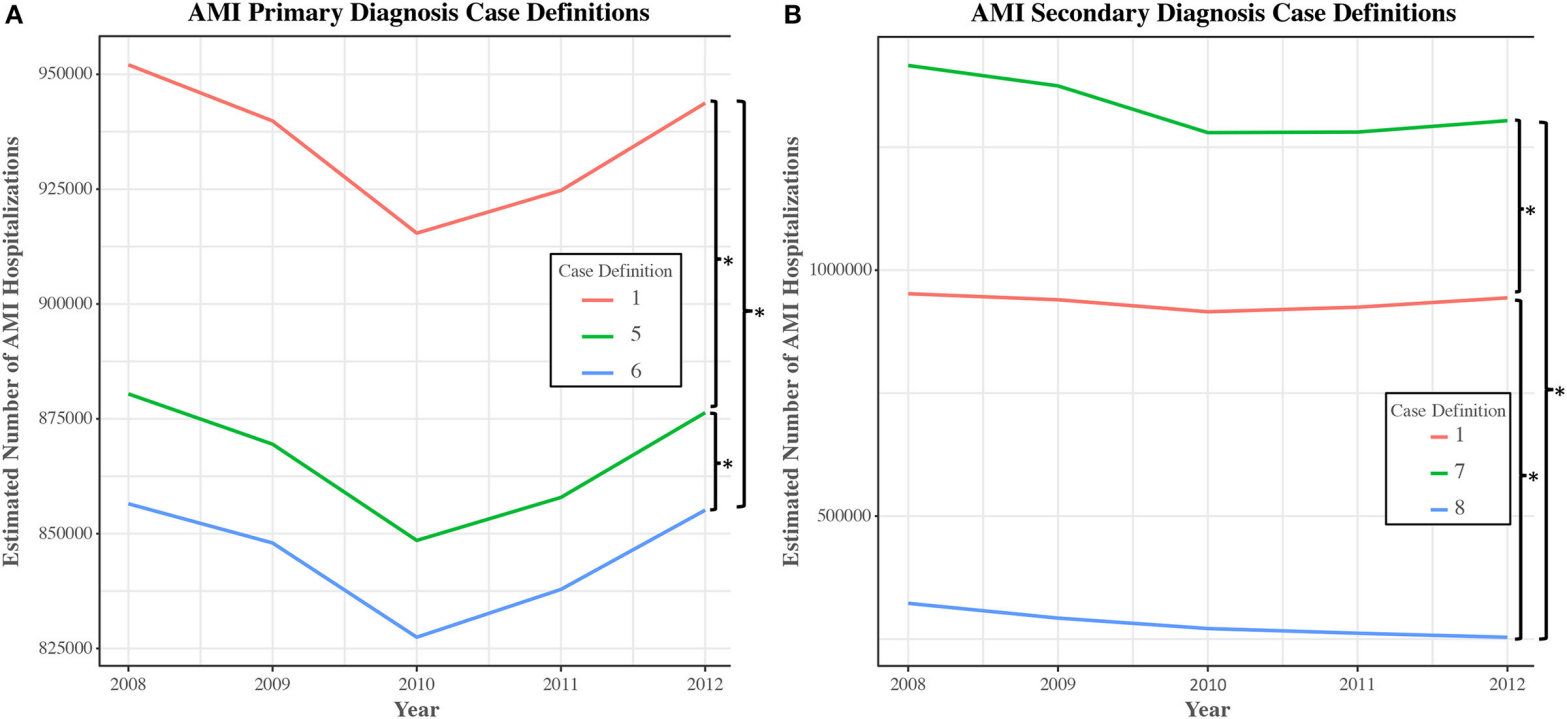
Number of discharges for selected unique case definitions for acute myocardial infarction in the national inpatient sample, 2008–2012. National-level trends in the number of hospital discharges were obtained using NIS data from 2008 to 2012. Estimates and trends of AMI (acute myocardial infarction) incidence were compared across the most common case definitions used to establish AMI as a primary **(A)** or secondary diagnosis **(B)**. Case definitions were extracted from the study cohort of articles. Of note, utilization of Case Definition #8 leads to an impression of a gradual reduction in AMI incidence over time, a pattern not observed with other case definitions ([β ± SE] −0.049 ± 0.008; *P* for difference in slopes < 0.001). *P < 0.001.

Upon reintroduction into the NIS for data years 2008–2012, different ICD-9-CM case definitions used to establish primary diagnoses for AMI in the study sample were found to yield statistically significant differences in estimates of AMI hospitalizations. Case definitions #1–4 each yielded 978,724 hospitalizations, #5 yielded 906,754, and #6 yielded 884,041 (#1 vs. #5: RR = 1.079, 95% CI [1.077–1.081], *P* < 0.001; #1 vs. #6: RR = 1.107, 95% CI [1.105–1.109], *P* < 0.001; #5 vs. #6: RR = 1.026, 95% CI [1.025–1.026], *P* < 0.001) (Figure 4A). Trends in AMI hospitalizations did not vary significantly between primary case definitions for the period studied.

Similar findings were uncovered in an analysis of selected case definitions used to establish AMI as a secondary diagnosis (#1 vs. #7: RR = 0.703, 95% CI [0.697–0.708], *P* < 0.001; #1 vs. #8: RR = 3.334, 95% CI [3.279–3.390], *P* < 0.001; #7 vs. #8: RR = 4.745, 95% CI [4.668–4.824], *P* < 0.001) (Figure 4B). In this case, trend analysis revealed that utilization of case definition #8 would lead to an impression of a gradual reduction in AMI hospitalizations over time, a pattern not observed with other case definitions ([β ± SE] −0.049 ± 0.008; *P* for difference in slopes < 0.001).

### Adherence to AHRQ Methodological Standards in Cardiac Research Using the NIS

Of the population of included articles, 262 (58.9%) included the necessary elements to permit evaluation by 5 of the required research practices, 175 (39.3%) could be evaluated by 6, and 8 (1.8%) could be evaluated by all practices. There was variable adherence to AHRQ methodological standards. 71 (16.0%) studies adhered to all of the research practices assessed. 144 studies did not adhere to one of the standards, while 139 were non-adherent to two standards (Table 3). 91 studies did not adhere to 3 or more of the required research practices, of which 78 (17.5%) failed to adhere to 3 practices, and 13 (2.9%) failed to adhere to 4 or more practices. Temporal analysis revealed an unchanged rate of non-adherence in cardiac literature over the past decade.

**Table 3:** Total number of instances of non-adherence to AHRQ methodological standards in cardiac literature using the national inpatient sample.

**No. of Instances of non-adherence**	**Overall (*n* = 445) No. (%)**	**Impact factor < 10 (*n* = 421) No. (%)**	Impact factor ≥ 10 (*n* = 24)^*^ No. (%)
0	71 (16.0)	64 (15.2)	7 (29.2)
1	144 (32.4)	132 (31.4)	12 (50.0)
2	139 (31.2)	135 (32.1)	4 (16.7)
3	78 (17.5)	77 (18.3)	1 (4.2)
≥4	13 (2.9)	13 (3.1)	0 (0)

* *P = 0.032 (χ^2^ test for comparison)*.

Taken together, higher-impact publications exhibited fewer instances of non-adherence to AHRQ methodological standards than did articles published in lower-impact journals (median, 1 [interquartile range (IQR), 0–1] vs. 2 [IQR 1–2], *P* = 0.002). This was driven primarily by superior adherence to required statistical methods (practice 6) in high-impact publications (*P* < 0.001), however a non-statistically significant trend in practice 1 also favored high-impact articles (Table 4). There was strong inter-rater reliability (κ statistic = 0.87) for abstraction of data concerning adherence to each of the aforementioned research methods.

**Table 4:** Instances of non-adherence to specific AHRQ methodological practices in cardiac literature using the national inpatient sample.

**Methodological practice**	**Overall No./Total (%)**	**Impact Factor < 10 No./Total (%)**	**Impact factor ≥ 10 No./Total (%)**	***P*** -value^*^
1: Recorded hospital events as patients	170/445 (38.2)	167/421 (39.7)	3/24 (12.5)	0.056
2: Performed state-level analyses	10/445 (2.2)	8/421 (1.9)	2/24 (8.3)	0.672
3: Performed hospital-level analyses after 2011	9/58 (15.5)^†^	9/51 (17.6)	0/7 (0)	1.0
4: Performed physician-level analyses	12/445 (2.7)	9/421 (2.1)	3/24 (12.5)	0.154
5: Employed non-specific secondary diagnosis codes	159/445 (35.7)	155/421 (36.8)	4/24 (16.7)	0.350
6: Used statistical methods that did not account for the complex survey structure of the NIS	250/445 (56.2)	247/421 (58.7)	3/24 (12.5)	0.0005
7: Did not adjust for major transition periods (1997–1998, 2011–2012) for trend analyses	102/122 (83.6)^‡^	94/114 (79.8)	8/8 (100)	1.0

* *P value computed via Fisher exact test with Bonferroni correction*.

† *58/445 studies performed hospital-level analyses*.

‡ *122/445 studies performed trend analysis involving major transition periods in the NIS (1997–1998 or 2011–2012)*.

## Discussion

In this 20-year observational study of 445 articles using data obtained from the NIS, considerable variation in ICD-9-CM code selection was observed for several major cardiac diagnoses and procedures, including AMI, HF, AF, PCI, and CABG. Off-target codes were frequently identified in the included articles. The number of different case definitions published per year was found to be increasing with time, driven largely by the significant increase in articles per year, possibly suggesting increasing standardization in the use of case definitions. Additionally, non-adherence to other established AHRQ methodological directives was found to be prevalent in cardiac research using the NIS, concordant with prior estimates (8). The overall study approach and observations are summarized in Supplementary Figure 1.

In a real-world simulation, the use of different case definitions derived from the study sample produced significantly different estimates of AMI hospitalizations and trends over time, highlighting that variation in code selection has the potential to influence study outcomes and generalizability. In the analysis of primary AMI case definitions, the observed variability in the number of hospitalizations was ultimately tied to whether the study authors highlighted any particular episode of care for AMI. Episode of care codes, added to ICD-9-CM for AMI in 1989 and denoted by the identity of the fifth digit, define the phase of treatment for clinical services. “410.x1” refers to the initial episode of care, namely the clinically-related services for one encounter during the acute phase of treatment for AMI, while “410.x2” refers explicitly to care received during a subsequent episode of care, hence does not capture index hospitalizations. “410.x0” refers to an unspecified episode of care. In this study, the majority of primary case definitions for AMI included codes for subsequent episodes of care. Given these non-index hospital admissions for MI may have distinct risk and cost profiles, their inclusion may confound the generalizability of epidemiologic, mortality, and cost estimates for care generated by these studies when compared with analyses restricted to index AMI hospitalizations alone.

In the analysis of secondary case definitions, variability was instead observed with respect to the diagnosis codes included. Specifically, the inclusion of “411” in case definition #7—the parent code for unstable angina—aberrantly increased estimates of AMI hospitalizations, while the exclusion of “4107”—the category code for NSTEMI—in case definition #8 decreased estimates of AMI hospitalizations over the simulated interval relative to other secondary AMI case definitions.

Finally, it is also important to note that not all coding variation produced different epidemiological estimates. Despite differences in the number and content of codes used, case definitions #1–4 (accounting for approximately 70% of all AMI case definitions observed in this study) produced identical estimates of AMI hospitalizations. This is likely a result of redundancy in the mechanism for how diagnoses and procedures are identified when using the NIS. As an example, AMI case definition #4 included only “410”—the non-billable category code that includes all billable ICD-9-CM codes for AMI—whereas case definition #2 included all of the possible billable ICD-9-CM codes for AMI. Importantly, this suggests that not all qualitative coding variation is quantitatively important: different case definitions may yield the same epidemiologic estimates.

Within this sample, off-target coding was observed in 8.8% of studies, however the prevalence was highly variable by the particular diagnosis and procedure examined; 1.5% of CABG articles cited off-target codes, compared with 30% of articles citing codes for unstable angina. Among the most dramatic of these examples lies in the articles citing codes for AF, 9.5% of which included codes for atrial flutter. Given AF and atrial flutter have different risk factors, pathophysiologic mechanisms, and therapeutic interventions, the inclusion of atrial flutter codes alongside atrial fibrillation codes may confound analyses focusing on hospitalization, readmission rates, cost analyses, stroke risk, or analyses of associated risk factors. This has importance for the generalizability of studies using, vs. not using, atrial flutter codes. To ensure accuracy, generalizability, and avoid confusion amongst readership, authors should ensure concordance between their desired diagnoses and associated diagnosis codes.

Collectively, these data extend existing concerns regarding the generalizability of published studies using the NIS (2, 8). Previous studies have observed coding variation for AMI and HF, although were largely restricted to a focus on code validation or limited by the inclusion of case definitions reported by articles using multiple administrative datasets (29, 30). In one analysis, estimates of AMI case volume and in-hospital mortality were found to vary between the most commonly used case definitions for AMI found in articles published in high-impact journals, provoking the authors to conclude that the consistent use of a single validated case definition for AMI (e.g., “410”) may improve generalizability between studies (29). Another analysis involving the Canadian National Hospital Discharge Abstract Database revealed similar variation in the use of ICD-9 and ICD-10 codes for HF, potentially explaining the observed variability in results across the included studies (30). The present analysis builds upon this work, showing that coding variation is persistent, progressive, and pervasive for major cardiac diagnoses and procedures in a single heavily used dataset representing the United States population.

These data also bear special implications for administrative dataset-based cardiac research. The NIS has been appropriated to study an ever-increasing purview of topics, ranging from disease epidemiology and healthcare utilization, to evaluating the efficacy of national-level policy interventions (31). Coding variability, as well as off-target coding, have the potential to confound the generalizability of these analyses. For instance, we found that inclusion of codes corresponding to HF with comorbid chronic kidney disease (“404x3”) was frequent in studies using the NIS, present in 41.1% of all HF case definitions. Targeting this population may have significant implications, given it is burdened by higher mortality and rehospitalization risk (32, 33).

Reasons for the observed coding variation could be manifold, including erroneous code selection, incorrect documentation, reporting and publication bias, responses to iterations in ICD-9-CM coding protocol, or intentional targeting of different populations without clear documentation to this effect. Beyond these, a significant challenge that remains is the absence of consensus guidelines for code selection for major cardiac diagnoses and procedures. Researchers utilizing the NIS have numerous and often divergent sources from which to obtain candidate codes. For AMI, differences remain between coding schema recommended by code validation studies (29), those curated by the HCUP (11), and those endorsed by clinical guideline-producing and quality improvement groups (5, 26, 28). Similar considerations exist for other cardiac diagnoses and procedures.

Ultimately, to maximize the comparability of studies using administrative data, this analysis highlights the need for greater standardization in administrative code selection for cardiac diagnoses and procedures. As an example, the American College of Cardiology and American Heart Association have recommended the use of specific ICD codes for evaluation of AMI-related performance measures. Similar national or international consensus standards could be developed for code selection, wherein case definitions with the most optimal performance characteristics (e.g., the ICD-9 code “410” for AMI) for cardiovascular conditions or procedures are recommended for administrative research applications across databases. Greater editorial oversight, as well as more explicit direction from database curators, may be needed to ensure adherence to best coding practices. In addition, greater clarity is needed in the reporting and rationale for code selection by study authors using the NIS, especially in cases where the case definitions used deviate from AHRQ-developed or other validated coding schema. The necessity of these measures is emphasized by the expanding use of ICD-10-CM, which offers a greater number of codes (34, 35).

Greater editorial oversight may also be needed to ensure adherence to database-specific methodological standards. In this study, 84% of the included articles exhibited non-adherence to at least one of the research practices required by the AHRQ. More than half of the articles did not report the use of appropriate statistical methodologies to account for the complex survey structure of the NIS, and over 80% did not report the use of appropriate statistical weighting techniques to account for changes in sampling structure for major NIS transition periods. Overall, these results are similar to a prior analysis evaluating methodologic adherence in sample of all studies published using the NIS (8), highlighting the importance of educating prospective users of administrative datasets in the use of the most appropriate research practices. Tools that address the unique structural elements of each dataset and provide explicit methodological guidance, such as the HCUP-NIS Checklist, represent a hopeful step forward (14). As an example, the use of non-specific secondary diagnosis to infer in-hospital events was seen in greater than one-third of articles in this study. To avoid these issues, NIS curators have recommended the use of either (1) specific ICD codes that indicate complications (e.g., 9954—shock due to anesthesia), or (2) AHRQ's validated patient safety indicators (14).

Several important limitations must be addressed. First, we conducted mostly qualitative review restricted to a single dataset, hence did not evaluate all case definitions for epidemiological equivalence within the NIS, nor did we evaluate the performance of observed case definitions within different datasets. Determining whether similar trends, as compared with AMI coding in this study, are observed for other cardiac and non-cardiac diagnoses and procedures within the NIS and other databases is an important focus of future research. Second, given our intention to conduct a pragmatic study based on all-comers, we did not explicitly address variation between validated and unvalidated coding schema for cardiac diagnoses and procedures. Third, as ICD-9-CM coding schema are dynamic, some of the variability observed in this study would be expected as a result of appropriate responses to iterations in coding protocol. However, given the vast majority of the articles included in the study were published after the most recent ICD-9-CM diagnosis- or procedure-specific update (e.g., AMI codes last updated in 1989), and that codes from older ICD-9-CM versions were often included in modern analyses, we feel this has little impact on the conclusions herein.

## Conclusions

In this study of cardiac research using the NIS, substantial variation in code selection for several major cardiovascular diagnoses and procedures was observed, and non-adherence to required research practices was widespread. This study emphasizes the need for consensus protocols for code utilization concerning major cardiac diagnoses and procedures. Prospective users of administrative data for research purposes should be explicit about the rationale for code selection for diagnoses and procedures, avoid off-target coding, and ensure adherence to research practices that account for unique database design elements.

## Data Availability Statement

The raw data supporting the conclusions of this article will be made available by the authors, without undue reservation.

## Ethics Statement

The studies involving human participants were reviewed and approved by Institutional Review Board, UT Health San Antonio. The use of NIS data for this study was exempted from further consideration by the UT Health San Antonio's institutional review board as the data are de-identified. Written informed consent for participation was not required for this study in accordance with the national legislation and the institutional requirements.

## Author Contributions

JO, JM, and AP: concept and design. JO: drafting of the manuscript. JA-G, JM, and AP: critical review of the manuscript. JO, BH, and JM: statistical analysis. JM and AP: administrative, technical, or material support. All authors acquisition, analysis, or interpretation of the data.

## Funding

JO was funded by an American Heart Association Student Scholarship in Cardiovascular Disease. AP was funded by a grant from the Freeman Heart Association.

## Conflict of Interest

The authors declare that the research was conducted in the absence of any commercial or financial relationships that could be construed as a potential conflict of interest.

## Publisher's Note

All claims expressed in this article are solely those of the authors and do not necessarily represent those of their affiliated organizations, or those of the publisher, the editors and the reviewers. Any product that may be evaluated in this article, or claim that may be made by its manufacturer, is not guaranteed or endorsed by the publisher.
