# Supplementary figures and images for: Coding Variation and Adherence to Methodological Standards in Cardiac Research Using the National Inpatient Sample
Source: Front Cardiovasc Med. 2021 Nov 2;8:713695. doi: 10.3389/fcvm.2021.713695 (PMC8592936; doi:10.3389/fcvm.2021.713695)

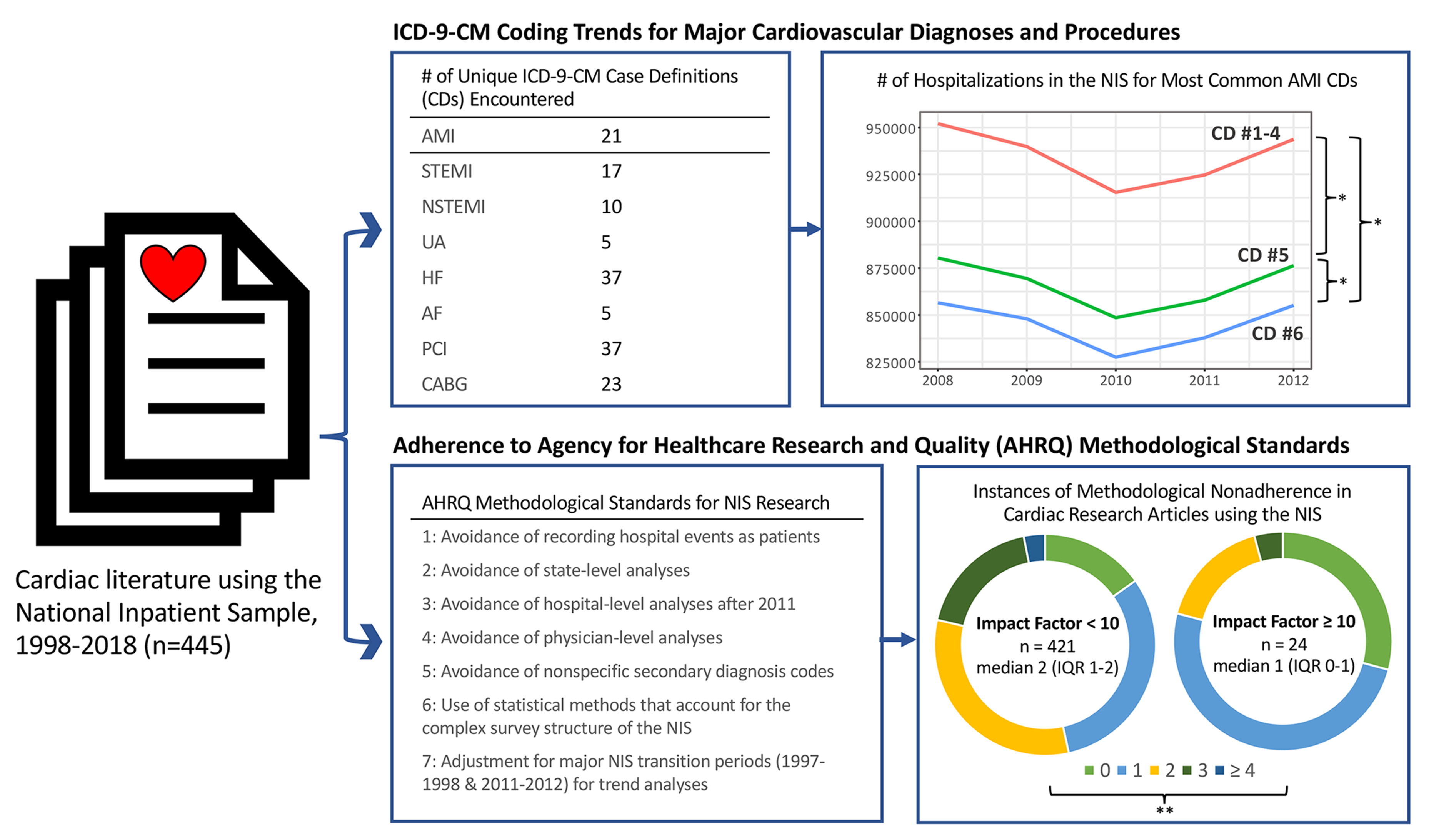

Supplement: Supplementary file 1 [file Image_1.TIF]
